# *STOP COVID-19 CA:* Community engagement to address the disparate impacts of the COVID-19 pandemic in California

**DOI:** 10.3389/frhs.2022.935297

**Published:** 2022-11-30

**Authors:** Alejandra Casillas, Lisa G. Rosas, Savanna L. Carson, Allison Orechwa, Gemma North, Mona AuYoung, Gloria Kim, Jesus A. Guereca, Christian B. Ramers, Nancy J. Burke, Claudia G. Corchado, Sergio Aguilar-Gaxiola, Ann Cheney, Borsika A. Rabin, Nicole A. Stadnick, William Oswald, Abby Cabrera, Dara H. Sorkin, Frank Zaldivar, Wennie Wong, Anusha S. Yerraguntala, Stefanie D. Vassar, Aziza Lucas Wright, Donna L. Washington, Keith C. Norris, Arleen F. Brown

**Affiliations:** ^1^Division of General Internal Medicine and Health Services Research, Department of Medicine, David Geffen School of Medicine, UCLA, Los Angeles, CA, United States; ^2^Department of Epidemiology and Population Health, Stanford School of Medicine, Stanford, CA, United States; ^3^Division of Primary Care and Population Health, Department of Medicine, Stanford School of Medicine, Stanford, CA, United States; ^4^Southern California Clinical and Translational Science Institute, University of Southern California, Los Angeles, CA, United States; ^5^Scripps Health, San Diego, CA, United States; ^6^Laura Rodriguez Research Institute Family Health Centers of San Diego, San Diego, CA, United States; ^7^Division of Infectious Diseases, Department of Medicine, University of California San Diego, La Jolla, CA, United States; ^8^Public Health Department, University of California, Merced, Merced, CA, United States; ^9^Cultiva La Salud, Merced, CA, United States; ^10^Center for Reducing Health Disparities and Community Engagement Program of the Clinical and Translational Science Center, Department of Internal Medicine, University of California, Davis, Sacramento, CA, United States; ^11^Department of Social Medicine Population and Public Health, School of Medicine, University of California, Riverside, Riverside, CA, United States; ^12^UC San Diego Altman Clinical Translational Research Institute Dissemination and Implementation Science Center, University of California San Diego, La Jolla, CA, United States; ^13^Herbert Wertheim School of Public Health and Human Longevity Science, University of California San Diego, La Jolla, CA, United States; ^14^Department of Psychiatry, School of Medicine, University of California San Diego, La Jolla, CA, United States; ^15^Child and Adolescent Services Research Center, San Diego, CA, United States; ^16^The Global Action Research Center, San Diego, CA, United States; ^17^Center for Excellence in Primary Care, Department of Family and Community Medicine, University of California, San Francisco, San Francisco, CA, United States; ^18^Department of Medicine, Institute for Clinical and Translational Science, University of California, Irvine, Irvine, CA, United States; ^19^Department of Pediatrics, Susan and Henry Samueli College of Health Sciences, University of California, Irvine, Irvine, CA, United States; ^20^Department of Preventive and Social Medicine, Charles R. Drew University of Medicine and Science, Los Angeles, CA, United States; ^21^South Central Prevention Coalition, Los Angeles, CA, United States; ^22^VA Greater Los Angeles Healthcare System, Los Angeles, CA, United States; ^23^Olive View Medical Center, Los Angeles County Department of Health Services, Sylmar, CA, United States

**Keywords:** COVID-19, community engagement (CE), health disparities, social determinant of health, community partnered participatory research (CPPR), state health policies

## Abstract

**Objective:**

To describe the early activities and lessons of the Share, Trust, Organize, Partner *COVID*-19 California Alliance (STOP COVID-19 CA), the California awardee of the NIH-funded multi-state Community Engagement Alliance (CEAL) against COVID-19. The Alliance was established to ensure equity in Coronavirus-19 disease (COVID-19) research, clinical practice, and public health for communities most impacted by the COVID-19 pandemic.

**Study setting:**

The STOP COVID-19 CA Alliance network of 11 universities and affiliated partner community-based organizations (CBOs) across California.

**Study design:**

Mixed methods evaluation consisting of an analysis of activity (August 2020 to December 2021) detailed in reports submitted by community-academic teams and a survey (August 2021) of academic investigators and affiliated community-based organization (CBO) partners.

**Data collection:**

We summarized activities from the 11 community-academic teams' progress reports and described results from an online survey of academic investigators and CBO partners in the California Alliance.

**Principal findings:**

A review of progress reports (*n* = 256) showed that teams fielded surveys to 11,000 Californians, conducted 133 focus groups, partnered with 29 vaccine/therapeutics clinical trials, and led more than 300 town halls and vaccine events that reached Californians from communities disproportionately impacted by COVID-19. Survey responses from academic investigators and CBO partners emphasized the importance of learning from the successes and challenges of the California Alliance teams' COVID-19 initiatives. Both academic and CBO respondents highlighted the need for streamlined federal and institutional administrative policies, and fiscal practices to promote more effective and timely operations of teams in their efforts to address the numerous underlying health and social disparities that predispose their communities to higher rates of, and poor outcomes from, COVID-19.

**Conclusions:**

STOP COVID-19 CA represents a new and potentially sustainable statewide community engagement model for addressing health disparities in multiethnic/multicultural and geographically dispersed communities.

## Introduction

California's population of 40 million residents is one of the most diverse and prosperous in the world (1–3), but the state is characterized by some of the most dramatic disparities in wealth and health (2, 3). The disproportionate impact of Coronavirus-19 disease (COVID-19) on racial or ethnic minorities and low-income communities across California provided stark evidence of these inequities (2–6). COVID-19 deaths among California's Hispanic or Latino/a/x (hereafter Latinx) residents were 30% higher than their representation in the state population, 33% higher in the African American or Black (hereafter Black) community, and 27% higher among American Indian and Alaskan Natives (hereafter AI/AN), and Native Hawaiian and Pacific Islander (hereafter NH/PI) communities (6–12). In addition to higher rates of COVID-19 infection, hospitalization, and death, these communities have also experienced lower vaccination and treatment rates than White peers in the state, at most points during the pandemic.

Factors rooted in the social determinants of health played an outsized role in risk and outcomes from COVID-19, among them: overrepresentation in essential worker jobs, little to no hazard pay or sick leave benefits, lack of personal protective equipment, limited access to health care, a higher burden of chronic conditions associated with magnified COVID-19 morbidity, digital divide disparities, poor quality of language-congruent information, the disproportionate impact of school closures, and multigenerational or overcrowded living conditions hindering social distancing or quarantining (13–17). Additionally, inconsistencies in COVID-19 information and outreach limited confidence in the scientific, medical, and government establishments (also stemming from historical and contemporary injustices) and enabled the proliferation of mis- and dis-information—to these most at-risk California residents (18–23).

Previous emergencies and natural disasters have demonstrated how locally and culturally tailored community engagement mitigate harms, build mutual understanding, and promote disaster recovery (24–26). As well, community-partnered planning for public health events or national disasters is associated with higher community trust and resilience (27–30). Community engagement activities facilitating preparedness and recovery include, but are not limited to: (1) community-tailored public health prevention and control in infectious outbreaks (e.g., social and behavioral change communication), (2) effective surveillance, testing and contract tracing through culturally-aligned interventions, (3) eliciting community needs for both clinical and non-clinical resources (e.g., rent relief, school re-opening, food assistance), and (4) timely and culturally-appropriate messaging. As such, the lack of robust community-partnered preparedness elements during the early days of the COVID-19 pandemic contributed to infection tracking errors, limited factual knowledge sharing, and augmented mistrust in populations at high risk for the disease (31–33).

To better advance a community-centered public health response to the COVID-19 pandemic, the National Institutes of Health (NIH) launched the Community Engagement Alliance (CEAL) in August 2020. With evidence pointing to the over-representation of racial and ethnic minority and low-income communities among COVID-19 infections, hospitalizations, deaths, and simultaneous under-representation of these same communities in COVID-19 vaccine and therapeutic trials, CEAL aimed to fund community-partnered research among states working to address such disparities in these medically underserved groups. The overarching goals of CEAL were to (i) understand specific factors that pertain to the disproportionate burden of COVID-19 in underserved communities, (ii) establish effective, community-engaged strategies to enhance education, awareness, access, and inclusion of these communities in research to advance the prevention and treatment of COVID-19, and (iii) address the misinformation, mistrust, and structural barriers slowing movement out of the pandemic (34).

**Figure 1:**
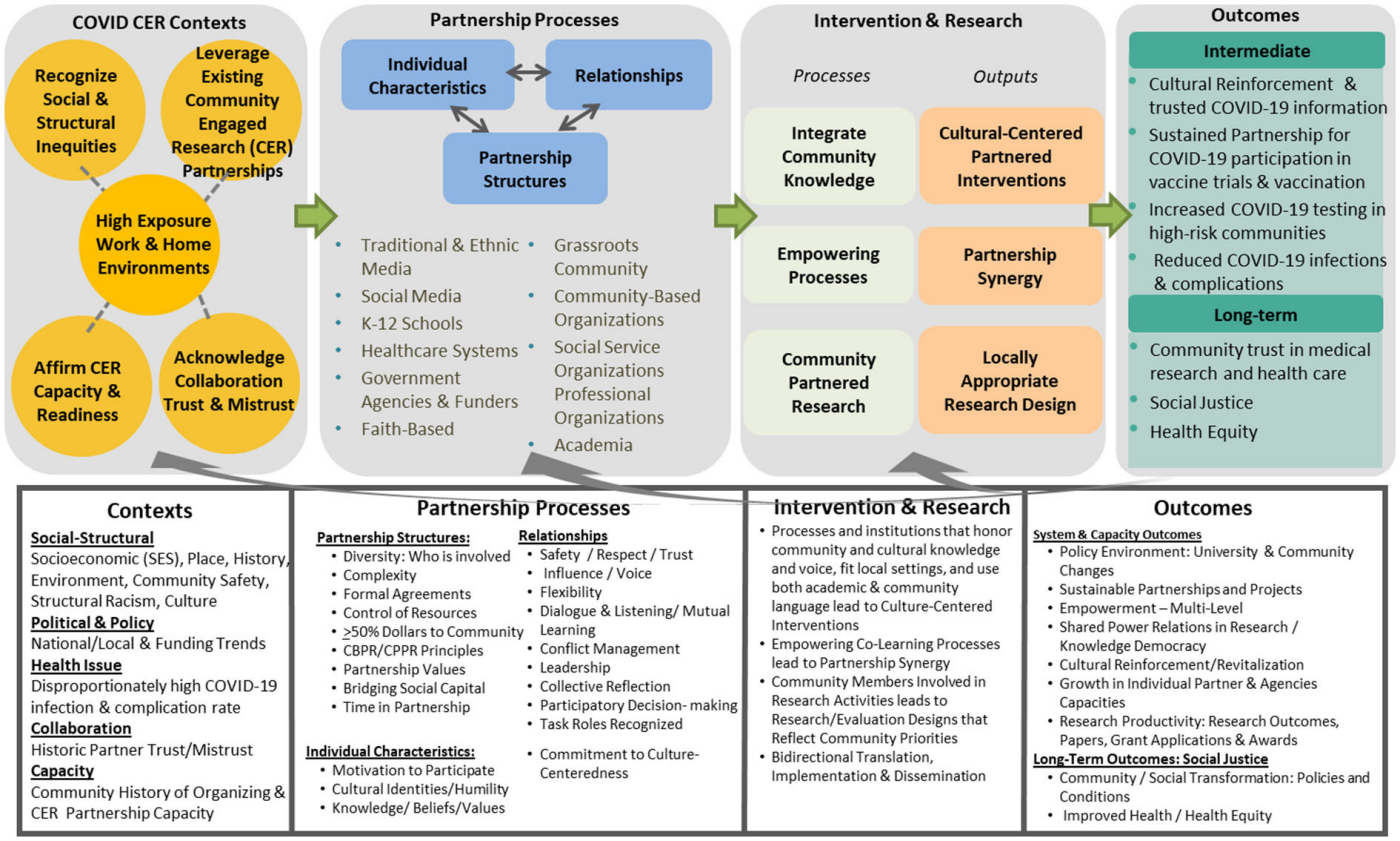
Conceptual framework on community engagement strategies to address California's COVID-19 disparities (modified from Wallerstein's community engagement conceptual framework) (35, 36).

The California state-based NIH CEAL consortium—the *S*hare, *T*rust, *O*rganize, *P*artner: the *COVID-19 C*alifornia *A*lliance (STOP COVID-19 CA)—was designed to promote COVID-19 research, clinical practice, and public health equity for the California communities hardest hit by the pandemic. The Alliance's aims were to (a) understand current community knowledge, and resource needs, (b) support recruitment into COVID-19 vaccine and therapeutic trials, and (c) develop evidence-based interventions to address COVID-19 disparities. The Alliance was guided by principles and strategies from Wallerstein's community engagement conceptual framework (35, 36), adapted to the COVID-19 pandemic (Figure 1). The model emphasizes important dynamics and outcomes in community-engaged research (CER): contextual and cultural centeredness, appropriate recruitment and retention strategies, and strengthened community. These also include partnering with community members to best contextualize an intervention for specific settings, integrating cultural values and practices to enhance sustainability when grant funding ends, and ultimately, democratizing science by valuing communities as equal contributors to the knowledge production process.

This summary describes Alliance activities and lessons learned from the perspectives of community-based organizations (CBOs) and academic investigators during the first year of STOP COVID-19 CA. We propose strategies for sustaining the Alliance to address ongoing community needs around COVID-19, future public health emergencies, and persistent social and health disparities.

## Methods

**Figure 2:**
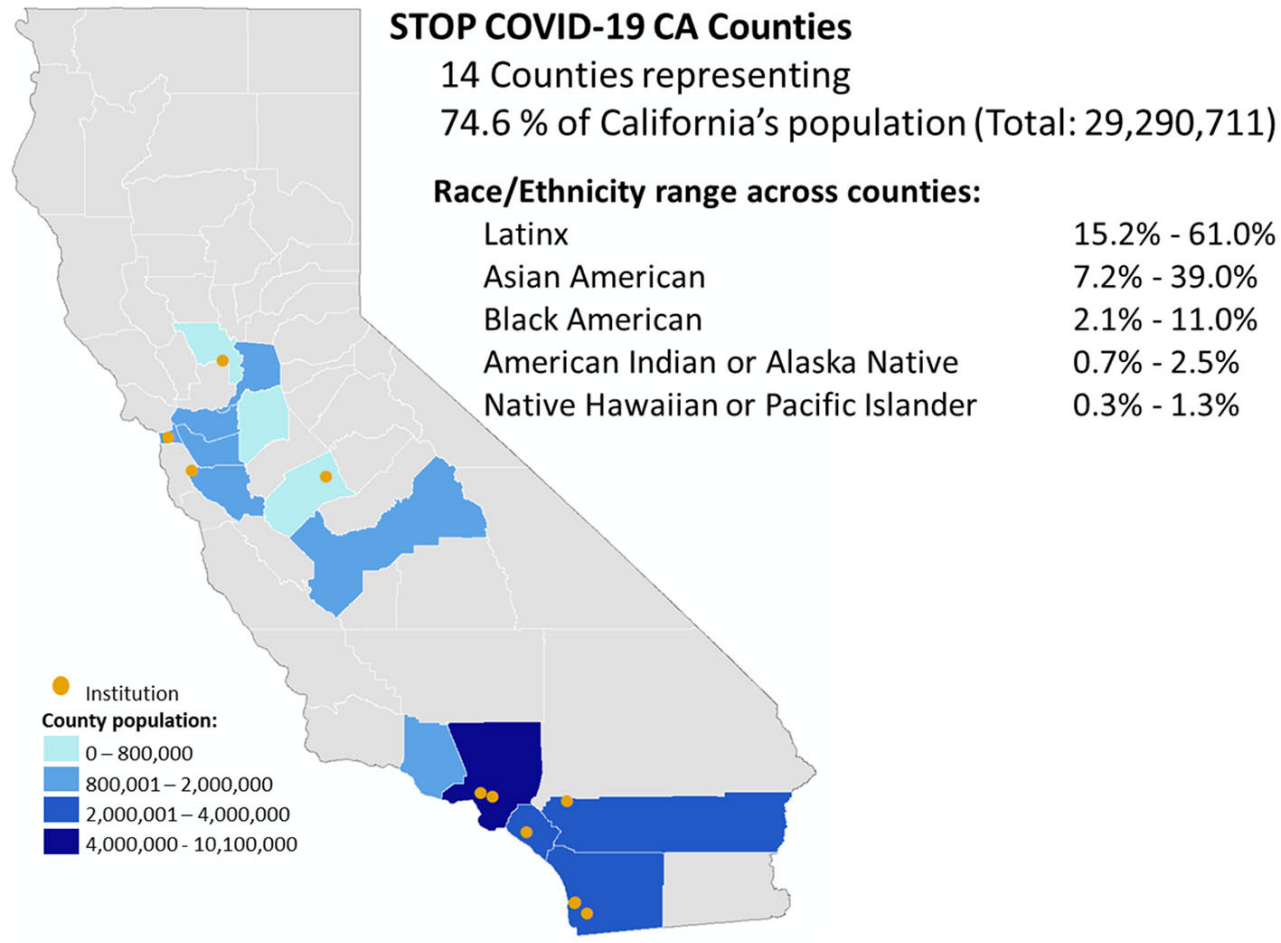
STOP COVID-19 CA academic-community teams showing county served across California.

Study Setting. The STOP COVID-19 CA Alliance consists of 11 teams; each team includes investigators and staff from an academic institution and representatives from their partnered network of community stakeholders (Table 1). The academic institutions include nine academic health centers [six University of California (UC) and three private institutions] and two universities (not affiliated with academic health centers). The Alliance spans the state of California (Figure 2), including all eight Clinical and Translational Science Awardees (CTSAs), three Research Centers in Minority-serving Institutions (RCMI) awardees (1 RCMI is part of a CTSA), and one non-RCMI minority-serving institution. Each of the 11 institutions has a community engagement program, representing longstanding relationships with CBOs, faith institutions, public health system stakeholders, and other agencies [e.g., the Veteran's Administration (VA), Federally Qualified Health Centers] central to the community-partnered infrastructure. Given the central role of community partners, the Alliance required that at least half of the overall California CEAL funding be distributed to community partner organizations. The 11 teams were organized to build local and statewide community-academic capacity to mitigate COVID-19 inequities *via* ongoing communication: In biweekly leadership meetings, academic and community core members shared updates on projects, new knowledge about the pandemic, emerging clinical and public health recommendations, and lessons learned in community settings. STOP COVID-19 CA faculty, staff, and community or stakeholder partners also collaborated in three biweekly primary working groups: Vaccine Hesitancy, Inclusive Participation in Research, and Communication.

**Table 1:** Key communities and individual activities for STOP COVID-19 CA teams (*n* = 11).

**Academic team**	**Key communities**	**Languages (other than English) for outreach and education**	**Main activities**
**San Diego State University**	Low-income safety net patients	Spanish	Motivational interviewing training
**Scripps**	Black, Latinx, NH/PI; Asian	Chinese; Spanish; Tagalog; Vietnamese	• Virtual town halls • Survey • Health fair outreach
**Stanford**	Latinx	Samoan; Spanish; Tongan	• Virtual town halls • Ethnic media training • Motivational interviewing training
**UC Davis**	Latinx; Farmworkers	Spanish; Cahuilla; Mixtec; Zapotec; Triqui	Radio outreach
**UC Irvine**	Parents of young children; Adolescents and Children	Spanish; Vietnamese	Focus groups
**UCLA**	Black; Latinx; NH/PI; Filipino; AI/AN; Veterans	Hawaiian; Samoan; Spanish; Tongan	• Coordination across the Alliance • Focus groups • Ethnic media training • Community Consultant Panel
**UC Merced**	Farmworkers	Hmong; Mixtec; Punjabi; Spanish; Zapotec	Focus groups
**UC Riverside**	• Black; AI/AN; Latinx • Farmworkers	Purépecha; Spanish	• Survey • Local artwork • Town Halls • Restorative Circles • CHW training • PSAs
**UC San Diego**	Immigrants and Refugees	Arabic; Dari; Kizagua; Spanish; Swahili	• Survey • Listening sessions
**UC San Francisco**	Black; Latinx; Chinese; Samoan; Young Adults	Cantonese; Spanish	• Focus groups • Community Advisory Board
**University of Southern California**	Black; Latinx	Spanish	• Focus groups • Survey • Virtual town halls • Public art campaign

COVID-19, Coronavirus Disease 2019; STOP COVID-19 CA, Share, Trust, Organize, Partner COVID-19 California Alliance; Latinx, Latino/a/x; Asian, Asian American; Black, African American or Black American; AI/AN, American Indian/Alaskan Native; NH/PI, Native Hawaiian/Pacific Islander.

### Study design

We produced a consolidated summary of activities from NIH progress reports and surveyed academic investigators and CBOs from the STOP COVID-19 CA community-academic teams.

### Review of statewide progress report

STOP COVID-19 CA submitted progress reports to the national NIH CEAL at regular intervals: weekly from September 2020 to May 2021, biweekly through December 2021, monthly and an annual report. Alliance teams' community and academic members reviewed and iteratively compiled all submitted reports (*n* = 256), developing consensus on categories that best captured the work and accomplishments of each team. Five categories were identified: (1) engaged research partnered with multiethnic communities historically underrepresented in research, (2) bi-directional informational outreach, (3) vaccine events, (4) tailored media messages, and (5) generation of new projects and funding. Once totals were compiled, each Alliance team was asked to review and validate the summary and provide final edits to its activities to ensure accuracy. The UCLA Institutional Review Board (lead site) determined that review for this evaluation was not required (IRB#20-001715).

### Statewide collaborative survey

In August 2021 (1 year into the Alliance), we conducted an online survey of academic and community stakeholders. The survey was co-developed by all teams, including statewide workgroups. It included demographic characteristics of the respondents, two Likert scale questions on impact and satisfaction with the Alliance, and 14 open-ended items on facilitators and barriers to implementation, strengths and challenges or limitations of the collaborative, community impact, lessons learned in community partnership, and workgroup-specific activities (see Supplementary material for survey). We requested at least one academic and one CBO response from each team, but anyone in the Alliance was welcome to respond. At the request of the teams, we allowed multiple respondents to collaborate on a survey (i.e., community-academic partnered combined surveys). Multiple respondents from a single organization (academic institution or CBO) were averaged or combined to ensure equal weighting for each organization. Community partner respondents received a gift card ($25) for survey completion. One coder with PhD-level expertise in qualitative methods and analysis (SC) conducted reflexive thematic analysis (37, 38) in Atlas.Ti on the 14 open-ended items. For validation, preliminary themes were reviewed in an Alliance-wide meeting and vetted iteratively by co-investigators with expertise in qualitative analysis (AC, LGR, NS).

## Results

### Description of alliance activities

The Alliance's community-academic research partnerships designed and deployed multifaceted strategies to address Alliance aims, summarized as a whole in Table 2, and highlighted in examples below.

**Table 2:** STOP COVID-19 CA alliance activities up to December 2021.

**California alliance activity**	**Number of events across all partnered teams**	**Number of teams participating in activity**
Research partnered with multiethnic communities		
Focus groups (number of participants)	133 (780)	9
Surveyed individuals	11,825	9
Stakeholder/leadership interviews in community	1,145	7
Vaccine/therapeutic clinical trials supported	29	8
Research/policy/public health briefs/reports	10	5
Scientific manuscripts published or in preparation	17	8
Bi-directional informational outreach		
Town halls (number of participants)	201 (58,249)	11
Health fairs (number of participants)	51 (52,286)	4
Restorative/healing circles (number of participants)	7 (71)	2
Educational programming, workshops (number of participants)	288 (16,850)	9
Promotoras participating in Alliance	353	8
Promotoras trained in COVID-19 information and messaging	468	5
Vaccine events		
Vaccine events and “pop-up clinics” (# vaccinated on-site)	130 (7,537)	7
Other vaccine supports (# people assisted with vaccine appointment, transportation)	11 (8,895)	3
Materials distributed at these events (e.g., PPE, food boxes, personal hygiene, toys)	3,722	2
Tailored media/messages developed		
Film/video creative content unique products (number of viewers)	699 (4,018,125)	5
TV segments	185	6
Radio segments (estimated listenership)	30 (471,600)	6
TV/Radio PSAs (estimated viewership/listenership)	63 (3,554,852)	5
Newspaper articles	17	6
Ads on banners/bench/digital/radio/TV (number reached)	2,158 (29,013)	4
Social media posts (number of reactions, e.g., “likes” or comments)	819 (3,317,904)	9
Websites (number of website views)	15 (38,083)	6
Number of accounts reached through email, text, app messaging (e.g., Google Voice, WhatsApp)	432,712	2
Number of languages covered for all new media	19	
New funding linked to STOP COVID-19 CA		
New grants on Alliance-related projects (separate from CEAL)	12	7
New grant funding received linked to CEAL	$32,311,299	5

COVID-19, Coronavirus Disease 2019; STOP COVID-19 CA, Share, Trust, Organize, Partner COVID-19 California Alliance; PSAs, Public Service Announcements; CEAL, Community Engagement Alliance; PPE, personal protective equipment.

#### Community-based data gathering to identify and address tailored needs

The Alliance allowed for trusted entry into unique communities at high risk for COVID-19 infection and impact, facilitating teams' access to real-time information about evolving needs. Partnered community-based research included assessing community needs, identifying trusted messengers, and understanding concerns about, interest in, and access to vaccination. Traditional academic outputs, such as research papers and policy briefs, were developed and disseminated collaboratively (39–44). Additionally, these results from focus groups, vaccine attitude surveys, community engagement events, and town halls were also used (39–44) to collaboratively inform and impact public health campaigns (21, 42, 45) and community-based outreach through CBOs, faith networks, clinic consortia, and other trusted local entities.

Targeted population-specific and community-based data gathering included racial and ethnic minorities, children, and low-income groups. For example, UC Irvine researched parent-child dyads to understand factors contributing to COVID-19 vaccine hesitancy and decision-making for parents and children. The USC team conducted focus groups to support a multimedia public health campaign called VaccinateLA (46) which informed culturally-tailored educational programming centering on Latinx and Black communities in East LA and South LA—now featured in animated YouTube videos, social media, local football games, TV (also in Spanish-Univision), and used by governmental agencies at the local, state, and national levels. UC Davis partnered with Radio Bilingue (47–50), a radio station broadcasting in Spanish, Mixteco, Zapoteco, and Triqui languages in the Central and Salinas Valleys to understand gaps in COVID-19 knowledge of monolingual farmworkers (a population more readily reached by radio vs. other media, particularly during working hours). Subsequently, the team produced broadcasts and hosted community events for farmworker constituencies on the availability of rapid antigen testing, vaccines, testing/vaccine integration, and implications for families and children.

Overall, eight Alliance teams directly partnered with COVID-19 vaccine and therapeutic clinical trials to develop community-centered protocols for COVID-19 research. The UCSF and UCLA teams organized community advisory boards in collaboration with local COVID-19 vaccine trials to diversify recruitment and outreach. Advisory boards improved understanding of community concerns, preferences, and priorities for resources in their communities, participatory decision-making for vaccine resources, and clinical trial recruitment understanding of barriers to clinical trial participation and tailored protocols and materials to promote inclusion in research (51). The UCSF COVID Research Patient and Community Advisory Board, comprised of 29 diverse patients, community leaders, and other stakeholders, provided consultation to researchers on 21 studies. At UCLA, a deliberative community engagement approach was used to form a Community Consultant Panel consisting of diverse community experts from across LA County, clinical trial investigators and staff, and Alliance faculty and staff. The Community Consultant Panel collaboration helped three local vaccine trials achieve a minority participation rate of 69–74%, far higher than the national average. Across the Alliance, each team worked to support COVID-19 therapeutic trials, informing community members of available treatments and eligibility.

#### Partnered rapid deployment to bring COVID-19 resources to the community

Due to early limitations in COVID-19 testing, personal protective equipment, informational outreach, and other resources, many community-academic teams worked locally to identify and deploy priority resources. In the first months after vaccines became available, low-income and minority communities were prioritized for vaccines through the state's distribution plan but experienced shortages or lengthy waits at local vaccine distribution sites and reported hesitancy about getting the vaccine.

For example, in the Central Valley, UC Merced collaborated with local public health department and Federally Qualified Health Centers to bring vaccines and rapid testing to farmworker populations in local flea markets (Spanish-, Hmong-, and Punjabi-speaking communities). Nearby at UC Davis, the Organizations to Reduce, Advance, and Lead in Equity Against COVID-19 initiative (52) helped farmworkers in the Central Valley access community-based COVID-19 diagnostic tests, vaccines, and other health services. They partnered with labor contractors, faith-based organizations, county health departments, community centers, and other farmworker-centered organizations. From February 2021 to February 2022, 536 initiative locations provided 21,298 COVID-19 tests with a cumulative 11% positivity rate. As vaccines became widely available, vaccinations were integrated into these Alliance-led testing locations.

In San Diego, the Scripps team worked with UCSD and the county health department to turn a planned health fair in a medically underserved community into an impromptu vaccination site when vaccines doses became available 2 days before the event. Working with trusted community and academic leaders from the Scripps team, UCSD, and the county health department, the partners promoted the health fair, recruiting community members to attend. At the fair, the team then answered questions, addressed concerns, and vaccinated over 400 primarily Black, Latinx, NH/PI, and Asian American community members.

Stanford University worked with several community partners to build a “*Promotoras de Salud*” (community health worker) corps for door-to-door outreach to promote vaccine uptake. Community health worker capacity-building efforts focused on developing and sharing health education materials tailored for the local Latinx community, designing and disseminating training in motivational interviewing, and building a bank of frequently asked questions about vaccines and COVID-19 prevention.

In Los Angeles County and the Central Valley, UCLA and the State of California partnered with 34 CBOs for the *Get Out the Vaccine* project. Using a process similar to campaigns for increasing voter turnout in elections (*Get Out the Vote*), the team sought to reduce structural barriers to COVID-19 vaccine registration in zip codes with low vaccine rates and high COVID-19 morbidity. Unemployed or underemployed local residents were hired and trained as canvassers to conduct door-to-door outreach and provide resources to educate and register people for vaccine appointments. Canvassers also connected residents to social services for food, rental, and employment assistance. From May through December 2021, canvassers knocked on over 4.2 million doors and had conversations with approximately 2.4 million people, registering nearly 60,000 for vaccination.

#### Innovative models for community-engaged research

The urgency of the pandemic prompted our Alliance to better respond and partner with CBOs, resulting in new ways to engage communities. The UC San Diego Alliance team worked with the Global Action Research Center CBO, 10 grassroots community groups and two policy partners to co-develop a novel Theory of Change (ToC) process (39, 40). The ToC sought to reflect the preferences and priorities of the communities, related action items, and measures of success and served as the basis for their team's development of policy-focused products around vaccine uptake and participation in clinical trials.

In another example, the SDSU team, in partnership with a local Federally Qualified Health Center, used motivational interviewing to develop and deploy a remote personalized outreach intervention for COVID-19 prevention and decision support for COVID-19 vaccination. This personalized and brief outreach intervention tool is being tested and compared to a baseline remote outreach intervention that uses standard CDC and NIH communication resources.

Other models proposed and implemented by the community leads in our Alliance included Restorative Circles, a community-based intervention rooted in liberation theology, and adapted to mitigate the psychological harms of the pandemic on collective health. The UC Riverside team implemented Restorative Circles with Latinx immigrant communities in two forums: *promotoras* involved in COVID-19 testing and vaccination efforts, and parents of children returning to in-person learning. Mental health providers, a community-partner investigator, and a youth leader co-facilitated the circles engaging participants in sharing their stories and concerns. This work informed a subsequent funded project implementing a series of nine restorative circles in three Latinx or Indigenous Mexican immigrant communities in the Inland Southern California desert region.

#### Cross-site collaborations within the california alliance and across the national CEAL network

One of the unique assets of this Alliance was enabling team activities to extend beyond local municipalities, for state and national impact. As part of the collaborative, the Alliance funded new measures in the California Health Interview Survey to focus on socioeconomic implications of the pandemic and measures of prevalence of anti-Asian bias, based on local communities' input (53). As another example, the UC Riverside team worked with *promotoras* to engage the indigenous Latinx population, a community with concerns for (21, 54, 55) testing and vaccination sites— due to misinformation, fear of deportation, and lack of trust in institutions. The model placed *promotoras* from the focus communities at mobile testing sites and as contact tracers within the county's public health system to build trust (56). This *promotora* model was adopted by a Coachella Valley Health Equity Collaborative in the Inland Desert Region and utilized to deploy *promotoras* at vaccination sites across the entire southern California desert. UC Merced work with its partner, *Cultiva la Salud* and artist Lalo Alcaraz, to develop and disseminate culturally and linguistically appropriate COVID-19 animations and messages in Spanish, English, and Zapotec, in an effort led by the Arizona CEAL collaborative (57), and the national campaign COVIDLatino.org (58, 59). These materials were adopted by the California State Department of Public Health and further disseminated nationally and internationally (60–62).

### Alliance survey of academic and CBOs stakeholders

We obtained 34 survey responses with representation from all 11 community-academic teams, including 17 investigators at 11 institutions and 19 community partners at 17 community organizations (including at least one partner for each of the 11 institutions). Key themes by participants included facilitators and barriers to the development of the Alliance, perceived impact, and lessons learned (Table 3).

**Table 3:** Themes from the statewide alliance evaluation survey (*n* = 34) and select quotes.

**Category**	**Theme**	**Description**	**Exemplar quotes**
Facilitators	Existing partnerships supported the rapid implementation of pandemic community engagement	Universities relied on existing and longstanding partnerships for rapid implementation in the statewide initiative.	“*Because we've worked in [the} past with many of [university name] colleagues we have TRUST… this prior relationship and work experiences is the cornerstone of continued authentic, collaborative partnership.”*—CBO
	Reach to impacted communities was expedited and expanded through community partner networks	Existing trust and connections between community partners and their communities facilitated outreach to those disproportionately impacted by the pandemic for COVID-19 information, research, and clinical trials	• “*Our group of promotoras who [sic] have served as trusted messengers in their community and have been very essential in terms of community outreach/engagement. We have listened through different barriers different organizations have encountered in our communities and recognized how similar barriers have affected us, and have thus taken actions (i.e., helped individuals register for the vaccine, bring mobile clinics to communities, etc.).”*—CBO • “*The most effective technique we used was having trusted messengers to communicate honest and transparent messaging and communication to communities around the constant changes coming out of CDC… helped establish and strengthen the trust with our community members.”* —*Academic*
	Alliance brought opportunity for critical community-based funding	Community-directed funding was able to support critical community-based engagement and meet community-partner needs.	• “*Most participants are small non-profits with tight budgets and anything to pay for our time is a godsend.”*—CBO • “*It helped to provide some amount of compensation during a time of uneasiness, fear, and loss of work for our volunteers.”*—CBO • “*Energized the CBOs to jump in and join in this fight and strengthen the community network for dealing with challenges in COVID-19.”*—CBO
Barriers	Rapid application and implementation timelines	Difficult to build community engagement infrastructure to support a statewide initiative over the rapid application and implementation timelines.	• “*Lack of time to conceptualize and fulfill cross-state projects such as interventions, etc. (beyond surveys/focus groups). Minimal funding for staff. Did not have bilingual meetings for some partners. [Difficulty] keeping up to date on all the 11 teams projects/progress/next steps, including funding/budgets.”*—Academic • “*Time was a barrier - we had to launch project in record time.”*—CBO
	Evolving Funding and Community Priorities	Friction between national and local priorities (including pandemic challenges and community partner needs).	“*[Our challenges included] Identifying and engaging virtually (!!!) community advisory board members; national focus on vaccine trial participation versus outreach and vaccination (community priority was NOT the vaccine trial); Key barrier for engaging community members was competing priorities in their own personal and professional lives as a result of the pandemic.”*—Academic
	Bureaucracy in distributing Funding to Community Partners	Funder and university bureaucracies made it difficult to quickly disburse funds to community partners.	• “*Award occurred quickly, but there was red tape getting the contracts to [Univ.] and then more red tape getting the sub-contracts to my organization, so we'd done five months of work before we got paid. We are financially stable enough to weather that kind of delay, and we trust [Univ. name] enough to perform work and believe we'll get paid later, but it would be a hard stop for some smaller organizations.”*—CBO • “*The original promise of having funding spurred CBO to hire, and then they were financially strapped with the costs because we couldn't get payment to them fast enough. Nearly broke them!”*—Academic
Perceived impact	Strengthen Partnership Building in the Pandemic	Community-academic partnerships grew because of close working relationships during the project.	• “*I think we each understand more about one another due to the projects. CBOs understand who we are, and we (the university) understand more about what CBOs do and how they work. It's strengthened our partnership.”*—Academic
			• “*We have taken time to get to know each partner's work in more depth. We have gained a deeper understanding, appreciation, and trust in each other's work. This has led to being able to establish a true bi-directional learning system that facilitates learning from each other.”*—CBO
	Ability to leverage the statewide community-academic network to advocate for policy change	The network of universities and community partners could advocate for key policy changes in addressing COVID-19 inequities.	• “*[The Alliance] allowed for strong network communication, and new connections with policymakers, agency contacts (public health, etc.) on a local and statewide level to share research and policy recommendations! We were able to have conversations across community, academic, and policy/government in real-time for working through on-the-ground information, research, and policy needs… who [in turn] put plans in place to help mitigate barriers.”*—Academic • “*We participated in several statewide and national meetings in which we argued for the need to recognize the different experiences in rural areas.”*—Academic • “*Helped us to get on the radar of [local county health department] to disaggregate data further.”*—*CBO* • “*Tackling a pandemic requires an equity-based approach, and I have appreciated there being a space for our community to have their voices heard, especially at an institutional level… By giving our community a space to center their hesitancies and concerns, it has given us space to advocate their voices throughout the city.”*—CBO
	Mutually-beneficial resources and problem-solving	Bi-lateral sharing of resources for rapid turnarounds and outreach: (a) community partners provided community expertise for informing and tailoring interventions, (b) academics provided expertise for COVID-19 information, vaccines, and the latest research.	“*The statewide collaborative has been extremely beneficial in harvesting experiential learnings within the state teams and sharing them in order to strengthen local team activities… added value of being a space for joint problem solving and resource sharing.”*—CBO “*[The Alliance] has provided the funding and knowledge necessary to educate our communities on accurate information pertaining to COVID-19 and the vaccine. Additionally, it has provided a supportive community of professionals who have served as a supportive group working towards the same age–a–to stop the propagation of COVID and increase the number of those vaccinated.”*—CBO
Lessons learned	Cultural tailoring	Community partnerships were key for tailoring strategies and resources for a diversity of local populations.	“*Among the highest incidence of death from COVID are farmworkers… we are saving lives by providing reliable information in the languages that farmworkers understand through [CBO] a trusted messenger which is a Latino grassroots roots non-profit… we are on the air 24/7 with live radio shows and recorded messages educating our audience about COVID, prevention, protocols, and up to date best practices.”*—CBO “*Not only increased outreach and strengthened partnerships, but increased and strengthened cultural/community tailoring of information…we can say we increased engagement across the board and our work allowed for broad and diverse engagement and exposure to many communities, and more than ever before…”*—Academic
	Need to address social determinants of health	Inequity in social drivers (housing, healthcare, employment, and education) remains and leads to disparities in vaccination, testing, and clinical trial participation.	• “*Economic need for higher minimum wage, housing burdens in the LA area increased by the pandemic, support for caregivers, long-COVID impacts, additional vaccine outreach, potential for boosters, changing CDC/local guidelines and outreach, mental health, rebuilding community/personal bonds.”*—Academic
			• “*While food and rent assistance has been provided to community members due to COVID-19, this continues to be a major issue. Unfortunately, our communities were greatly affected and a month of assistance (which is what most families receive) simply isn't enough*.”—CBO • “*The collaborative can successfully address misinformation and hesitancy. However, the underlying disparities within housing, healthcare, employment, etc., need to be addressed on the policy level.”*—CBO
	Community engagement is critical throughout the process	Community priorities and needs change as the science unfolds and pandemic socioeconomic effects deepen. Consistent feedback and co-learning is essential to appropriate and resonant COVID-19 response	• “*Continued partnership for work allows community voices to be heard; Understanding how residents are thinking and making choices helps us all do better in communicating out the public health messages that resonate with concerns community residents struggle with.”*—CBO • “*The challenges were… the lack of appropriate outreach and educational material. Material was not published in enough languages, and the material was not relatable to many community members. Targeted communities need to be involved in the development of educational and outreach materials from the beginning.”*—CBO
	Longitudinal academic-community partnership, funding, and collaboration is needed to sustain efforts	Sustainment of trust and partnership between the medical, science, and public is needed	• “*Program recommendations are for continued collaboration by educational institutions, NIH, with authentic grassroots organizations who are trusted messengers among vulnerable populations; it should not be only during a pandemic.”*—Academic • “*For the community to trust the academics, a single project is not enough. The researchers have to come several times with several projects for the community to believe in them. It is important to know the culture and language of the communities.”*—CBO

CBO, Community-based organization; NIH, National Institutes of Health; COVID-19, Coronavirus Disease 2019.

#### Alliance implementation facilitators

Respondents emphasized that implementation of the Alliance was made possible by several factors. Given the rapid launch of the Alliance and its focus on local tailoring, many academic partners initially reached out to their existing, trusted partners to identify and develop Alliance activities. Additionally, community outreach to the most vulnerable populations was facilitated by individual and organizational partners embedded into the Alliance teams, who had both reach and trust within their communities. Finally, the recommendation to provide substantial funding to community partners for their role in addressing COVID-19 inequities was crucial to success, despite bureaucratic challenges, as discussed below.

#### Alliance implementation barriers

Respondents identified several barriers to the implementation of the Alliance. The rapid timeline to assemble the statewide team and a research proposal responsive to the global emergency conflicted with many important aspects of community partnered research, including a limited timeframe for meaningful community engagement in assessing real-time community needs and available resources, representation of heterogeneous community voices, and inclusion of the diverse needs and priorities of all partners. Many academic-community teams felt it was challenging to keep team members updated and conduct comprehensive strategic planning within the rapid funding windows. Each Alliance team was fortunate to have strong relationships with many community organizations, but described not having sufficient time to build relationships with other organizations relevant to this work due to the short application timeline.

The participants indicated that the priorities of the multiple stakeholders often differed, creating tensions between national and local priorities. For example, NIH CEAL funding priorities were initially focused on recruitment and inclusive participation in vaccine and treatment trials. In contrast, CBOs' priorities were to address the immediate needs arising from the pandemic- such as access to masks and cleaning supplies, trusted experts with accurate COVID-19 information, access to testing, and addressing pandemic-driven social determinants of health. In addition, policies and practices for sub-contract agreements at the institutional and federal levels created challenges in distributing funding and resources quickly to teams, especially community partner organizations. Respondents emphasized the need for academic-community funding mechanisms that allow up-front funding instead of standard cost-reimbursement models.

#### Perceptions of alliance impact

Participants described how the Alliance strengthened existing partnerships and created new important relationships, as needs surfaced and changed over time in response to evolving pandemic risk and emerging scientific and social developments (e.g., shifting from vaccine development to population-based vaccination). New connections were made with policymakers *via* the Alliance's state network, and created spaces where community partners could advocate for change in state vaccine campaign policies, like initiatives that would focus on enhancing access in rural and resource-scarce regions. The statewide network also leveraged local, regional, state, and national connections to share knowledge and advocate for community needs as new research findings emerged from across the Alliance. Additionally, the network provided a platform for sharing stories, materials, strategies, and collaborative problem-solving, with input from expert partners across the state facing similar local challenges.

#### Lessons learned

Given the diversity of California, respondents endorsed the importance of outreach suited to specific community preferences, needs, and desires. These consisted of: (i) clear communication in the language of the community, at the appropriate literacy level, and delivered through trusted messengers, (ii) translating scientific knowledge from academic partners into accessible lay language using visually appealing materials, and (iii) COVID-19 information and resources that addressed barriers related to the social determinants of health and the social context of a given community.

Evolving scientific evidence about COVID-19, its prevention and treatment, and its socioeconomic impact highlighted the importance of flexibility and responsiveness to changing community needs and concerns. For example, although the Alliance's initial priorities were to prevent COVID-19 infection and encourage participation in vaccine research, our community partners advocated for the need to address community concerns including housing, food insecurity, access to healthcare (including for depression), employment opportunities within COVID-19 research, as well as access to testing, vaccination, and clinical trials. Finally, community-academic partners endorsed the importance of longitudinal community engagement to develop community-relevant solutions that reduce health disparities— and not only in pandemics or other emergencies. Alliance participants emphasized the importance of preserving trust and continued bi-directional knowledge exchange between diverse, underserved, and under-resourced communities and the types of scientific, medical, and public health establishments in STOP COVID-19 CA.

## Discussion

STOP COVID-19 CA represents a new and potentially sustainable community engagement model for addressing disparities in multiethnic and multicultural, low-income communities geographically dispersed across California (41). This early-stage multi-method examination of the impact of the Alliance demonstrates the importance of leveraging both local community and academic expertise and the statewide infrastructure to influence outreach, research, and policy. The partners identified several facilitators and barriers to rapid implementation of the Alliance, identified beneficial outcomes, and highlighted important recommendations for addressing urgent and chronic public health needs facing vulnerable populations statewide. Among the key lessons were the importance of longitudinal community-academic relationships to address emerging issues and evolving community needs throughout a crisis, the need to ensure that the social determinants of health be centered in subsequent initiatives, and a call to reform and simplify funding processes for CBOs and other partners in community-academic research.

Alliance startup and operations were facilitated by the participation of teams with longstanding community-academic partnerships, leadership that included embedded community partners with an ability to expand reach to vulnerable communities, and the provision of a substantial proportion of the funding to community partners. These approaches have been identified as central to building novel collaborations that support multidisciplinary public health interventions (63–65). Several benefits from participation in the Alliance were described by stakeholders, many of which have also been identified in other community-based work during the pandemic (66–72). Alliance members reported expansion of community networks, broadened access to culturally specific COVID-19 messaging and vaccine outreach strategies, accelerated knowledge sharing by learning from the successes and challenges of other teams' initiatives, and leveraging the STOP COVID-19 CA network to reach local, state, and federal policymakers.

Despite the many benefits of the Alliance, our findings are also a call to action for investigators, clinical and public health leaders, funders, and local and state governments for how to ensure this model is improved and sustained to address the evolving COVID-19 pandemic health disparities across California (73, 74). One fundamental concern is that models like STOP COVID-19 CA and the more extensive CEAL network (despite a commitment to engaged scholarship) continue to center on academic perspectives, where community expertise is rarely or inequitably included (75–78). We must continue to re-conceptualize strategies for authentic community-engaged research that builds on community strengths and addresses the inherent power imbalance in current community-university partnerships (79, 80). Achieving this goal requires moving beyond inclusivity and toward equity (community “investigators” vs. community “partners”) in the funding, planning, implementation, evaluation, and dissemination of these efforts. It will be important to allocate sufficient time at the beginning of the funding period to co-create research, community engagement strategies, and outreach plans with existing grant-funded CBOs while building capacity for scholarship with new community partners.

Another prominent barrier highlighted by Alliance teams is the traditional approach to control and allocation of research funds. Generally, academic partners receive funding from NIH and subsequently distribute it to community partners with subcontracts. This process creates administrative burdens and delays for both community and academic partners. It inherently privileges academic experts over those with lived experience or who provide services, even though the latter perspective is essential to effective public health crisis responses. Bureaucratic challenges described included delays in paying CBOs, difficulty giving funding to new partners who were unanticipated at the time of budget preparation, and long and complex processes for completing or changing contracts and deliverables. To address these bureaucratic challenges, we must reconsider the current funding model to recognize that CBOs must hire and fund staff and projects quickly and move nimbly to address community needs. New models are needed to make funding more accessible to community stakeholders (81). One strategy may be to fund CBOs directly through performance-based awards (e.g., in four installments, with 25% upon signing the subcontract). Of note, the California Breast Cancer Research Program already provides separate contracts to academic and CBO partners from the funder (82).

Effective multidisciplinary dissemination of lessons learned within community-academic partnered strategies remains a challenge. As has been observed in prior research of community networks (83–86), the Alliance accelerated knowledge-exchange between community-academic teams. Future efforts could benefit from even more robust platforms and infrastructure to facilitate sharing and development of best practices, recommendations for dissemination of findings, and strategies to ascribe credit for community-driven activities and share lessons learned with key stakeholders such as policymakers, constituents, and media. There is also a need for venues to assist the rapid and effective sharing of findings with other state and national networks.

These analyses have some limitations. First, this summary and evaluation were conducted by Alliance teams; self-evaluation may be biased and heavily influenced by academic partner input. In addition, the categories of activities were developed by the writing team, best capturing the NIH progress reports used to compile data. It is possible that certain activities or initiatives by specific teams may not be accurately reflected here because they did not fit into existing reporting frameworks and were undercounted. However, we had each Alliance team review the summaries to verify the accuracy of the activities. Additionally, it is difficult to estimate our activities' total reach, including the secondary impact of this work and its partnerships.

Although the coronavirus outbreak has been called “*the great equalizer*” because we all experienced its impact in some way, the COVID-19 pandemic is more accurately described by the words of poet Damian Barr: “W*e are in the same storm, but not in the same boat*” (87). The people of California faced the virus, subsequent lockdown, and pandemic consequences from unequal starting points, and thus there is no uniform set of crisis responses that will be effective for all communities (88). To tackle these profound disparities, our health systems, research funders, and academic health centers must invest in and partner with communities, in order to build capacity for public health responses that are visibly authentic, trusted by community, and effective at promoting equity. STOP COVID-19 CA community-academic teams engaged members of high-risk populations in partnered research and tailored outreach to promote effective communication, increase vaccination and other prevention, and address pandemic-related social needs. We identified facilitators and barriers to the development and sustainment of the Alliance, benefits of this new statewide network, and recommendations for addressing COVID-19 in vulnerable populations statewide. STOP COVID-19 CA provides one such avenue forward— serving as a model for addressing future emergencies as well as the chronic public health and social disparities facing vulnerable populations statewide.

## Data availability statement

The raw data supporting the conclusions of this article will be made available by the authors, without undue reservation.

## Ethics statement

The studies involving human participants were reviewed and approved by UCLA IRB. Written informed consent for participation was not required for this study in accordance with the national legislation and the institutional requirements.

## Author contributions

AlC led the project analysis and primary writing and revisions for this manuscript. LR, NB, and SC led the qualitative analysis. AO, GN, MA, GK, JG, WW, and AY led the quantitative analysis. CR, CC, SA-G, AnC, BR, NS, WO, AbC, DS, FZ, AW, and DW provided key edits and revisions to the paper. SV provided key edits and designed tabled and figures, as project directors. KN and AB (co-PI's) were primary writers and provided key revisions to AlC. All authors are academic or community PI's/Co-I's on the STOP COVID-19 CA team. All authors contributed to the article and approved the submitted version.

## Funding

This paper was supported by the following funding NIH/NHLBI CEAL grant #21-312-0217571-66106L, NIH/NIMHD grant #P50-MD017366, and NIH/NCATS grant #UL1TR001881.

## Conflict of interest

Author CC was employed by Cultiva La Salud. Author AW was employed by South Central Prevention Coalition. Author MA was employed by Scripps Health. The remaining authors declare that the research was conducted in the absence of any commercial or financial relationships that could be construed as a potential conflict of interest.

## Publisher's note

All claims expressed in this article are solely those of the authors and do not necessarily represent those of their affiliated organizations, or those of the publisher, the editors and the reviewers. Any product that may be evaluated in this article, or claim that may be made by its manufacturer, is not guaranteed or endorsed by the publisher.
