# Supplementary material for: STOP COVID-19 CA: Community engagement to address the disparate impacts of the COVID-19 pandemic in California
Source: Front Health Serv. 2022 Nov 30;2:935297. doi: 10.3389/frhs.2022.935297 (PMC10012632; doi:10.3389/frhs.2022.935297)
Supplement: Supplementary file 1 [file Data_Sheet_1.PDF]

STOP COVID-19 CA

Demographics

Person (full name) filling out this survey (if filling out on behalf of multiple individuals or a site, please specify here):

[ ]

Name of affiliated University or Organization

[ ]

I am primarily affiliated with:

- ☐ A community organization / community representative
- ☐ A university

My university position is:

- ☐ Staff/administration
- ☐ Site PI
- ☐ Researcher/faculty
- ☐ [ ] Other

Which of the following describes your organization? You can choose more than one category.

- ☐ a. Health Care Organization (hospital, clinic, treatment facility, etc.)
- ☐ b. Faith-Based Organization (i.e., church, temple, etc.)
- ☐ c. Recreational Organization (i.e., YMCA)
- ☐ d. Social Service Organization
- ☐ e. Educational Organization
- ☐ f. Cultural Organization
- ☐ g. Other—please specify:

Populations served:

Services provided (check all that apply):

- ☐ a. Advocacy
- ☐ b. Arts
- ☐ c. Aging/Senior Citizens
- ☐ d. Community Services/Resources
- ☐ e. Education/Training
- ☐ f. Faith-based
- ☐ g. Health
- ☐ h. Homeless/Housing
- ☐ i. Justice / Re-Entry
- ☐ j. Legal Aid
- ☐ k. LGBTQ+
- ☐ l. Mental Health
- ☐ m. Nutrition/Food Security
- ☐ n. Social Justice
- ☐ o. Social services
- ☐ p. Substance abuse
- ☐ q. Youth/Children
- ☐ Other:

Email/contact (also used for community partners to receive giftcard for survey completion):

A. NIH CEAL / STOP COVID-19 CA Implementation

1. What were the barriers and facilitators to launching and implementing NIH CEAL / STOP COVID-19 CA quickly? Both *locally* and *statewide.*

2. Existing/New partnerships leveraged for implementation of NIH CEAL / STOP COVID-19 CA

What types of existing institutional and community programs/resources were leveraged to set up NIH CEAL / STOP COVID-19 CA quickly?

At your site, how long had the partnerships existed before NIH CEAL / STOP COVID-19 CA work started?

If these were newer partnerships, what strategies did you use to get the partnership running quickly and build trust (setting up MOUs, multiple meetings, etc.)?

3. What institutional barriers affected implementation or community partnership? How did you work around these barriers?

4. Funding *feasibility*, funding *limitations*, and funding impact on CBOs

What was feasible with the funding received?

What was limiting with the funding received?

How did this burst of funding impact CBOs?

5. Funding academic-community partnerships

Generally, how was the funding distribution structured?

What % of total funding received was given to community (partner CBOs, hiring from community, events, etc.)?

How did you get the funds to your partner(s) to support their work?

What worked and didn't work for community-directed funding?

6. What additional resources were offered or supplied to community partners (beyond the funding) to support partner participation?

B. Statewide and community-academic partnership collaboration

7. How have your academic-community partnerships evolved as a result of NIH CEAL / STOP COVID-19 CA?

8. What were the *strengths, benefits, and value added* of the multi-site statewide collaboration and the community-academic partnerships within the collaborative?

9. What were the *limitations* of the multi-site statewide collaboration and the partnerships within the collaborative?

10. How have you used materials, information, knowledge, or best practices you obtained from the NIH CEAL / STOP COVID-19 CA collaborative?

(Potential examples: strategic planning for COVID-19 community outreach/engagement, staff/partner/researcher education/development/capacity building, developing research protocols, development of products, advocacy/activism, etc.)

11. If you had a question, suggestion, or problem related to research for your academic/partner organization, how likely would you be to contact collaborators in NIH CEAL / STOP COVID-19 CA? **Why or why not?**

12. COVID-19 & mental health (within NIH CEAL / STOP COVID-19 CA Partnerships and within the Collaborative)

How has mental health come up in your partnerships and within the collaborative?

What strategies have you used to support your team at your site, within your partnership, or within the collaborative?

C. Community Engagement

13. How have academic-community partners and community engagement activities *been incorporated* into various projects, research, or other activities in NIH CEAL / STOP

COVID-19 CA? How have academic-community partners *feedback/input changed/advanced/broadened your activities* below?

a. COVID-19 Vaccine clinical trials, please specify why, how, who, what:

b. COVID-19 treatment/therapeutic clinical trials, please specify why, how, who, what:

c. COVID-19 information outreach, please specify why, how, who, what:

d. COVID-19 vaccine information outreach, please specify why, how, who, what:

e. COVID-19 testing or vaccination events, please specify why, how, who, what:

f. Research projects, please specify why, how, who, what:

g. Other, please specify why, how, who, what:

14. How are you evaluating community engagement in your work?

15. Do you feel your contributions were met with mutual respect, understanding, and trust within NIH CEAL / STOP COVID-19 CA?

	N/A: I had limited exposure to these groups	Strongly disagree	Somewhat disagree	Neither agree nor disagree	Somewh[illegible] agree
a. From academic partners	○	○	○	○	○
b. From community partners	○	○	○	○	○
c. From other collaborative members	○	○	○	○	○
e. From national teams (national workgroup members, NIH, etc)?	○	○	○	○	○
d. Comments on any answers from the above, please specify: [ ]	○	○	○	○	○

D. Community/Systems Impact

16. How has the NIH CEAL / STOP COVID-19 CA directly impacted your communities?

	N/A: I am unsure or not involved			Neither

	on this level in my role	Strongly disagree	Somewhat disagree	agree nor disagree	Somewha agree
a. Outreach/partnership to community partners	○	○	○	○	○
b. COVID19 Vaccine knowledge/understanding	○	○	○	○	○
c. Vaccination events	○	○	○	○	○
d. COVID-19 informational events	○	○	○	○	○
e. Research projects/outcomes	○	○	○	○	○
f. Brokered resources or leveraged partnerships for increased capacity	○	○	○	○	○
g. Capacity building: training, teaching, funding	○	○	○	○	○
h. Evaluated/identification of community interest/needs	○	○	○	○	○
i. Funding	○	○	○	○	○
j. Other, please specify:	○	○	○	○	○

17. Please share any comments on how the collaborative directly impacted your communities:

18. In what ways have you worked to build community capacity due to NIH CEAL / STOP COVID-19 CA ? Please explain.

19. How has your work within the collaborative led to policy/program recommendations or systems change?

20. What areas of COVID-19 recovery and relief still need to be addressed in your communit(ies)?

E. Assess lessons from CEAL for post-pandemic partnerships and research

21. Collaborative Sustainment

How can current collaborative partnerships be sustained?

What would help support sustainment of the collaborative?

22. What future functions of the collaborative do you foresee?

23. Academic-community partnerships - next steps & lessons learned

How can this collaborative inform future

academic-community partnerships?

How can we improve academic-community partners within the collaborative?

24. What other new projects/grants/works have occurred due to the collaborative?

25. Any other general comments/suggestions/feedback for the NIH CEAL / STOP COVID-19 CA collaborative?

F. Workgroup specific questions

VHWG Paper – AJ Paris Jackson, Evelyn Vazquez, & collaborators

26. For the communities you served, which groups appeared most hesitant or faced barriers to vaccine access? Please explain.

27. Addressing Vaccine Hesitancy

How did your team collaborate with others to address concerns or address barriers for this community?

Please describe any techniques or approaches your team engaged to address vaccine hesitancy or reduce barriers?

28. Were there times when changes to CDC guidelines or local policies created challenges for communities you worked with? If yes, please also describe any techniques or approaches your team engaged to adjust for changes to national/regional/local COVID-19 policy changes to support communities?

Community Health Worker / Promotores Paper – Christian Starks & collaborators

29. Did you partner, utilize, or harness community health workers/promotores during NIH CEAL / STOP COVID-19 CA ? If so, how so?

30. How are promotores using their unique expertise to sign up community residents to receive the SARS-CoV-2 vaccine? To encourage testing? How are promotores using their expertise to address vaccine hesitancy?

31. Would you be willing to answer further questions on your work or partnership with promotores in COVID-19 outreach or within STOP COVID CA? Y/N, other?

Restorative Justice Circles Paper – AJ Paris Jackson, Evelyn Vazquez, & collaborators

32. Did your team implement restorative circles? If so, how would you describe the experience? If so, what were the resources provided with the community (e.g. housing material, legal information, mental health support)

33. If you implemented restorative circles, describe how your community partnership was both equitable and inequitable?

34. If you implemented restorative circles, would you describe your partnership with a community group as restorative to both partners? Please describe how so or how not.

Powered by Qualtrics
